# *In Vitro* Activity of a Novel Antifungal Compound, MYC-053, against Clinically Significant Antifungal-Resistant Strains of *Candida glabrata*, *Candida auris*, *Cryptococcus neoformans*, and *Pneumocystis* spp.

**DOI:** 10.1128/AAC.01975-18

**Published:** 2019-03-27

**Authors:** G. Tetz, M. Collins, D. Vikina, V. Tetz

**Affiliations:** a TGV-Therapeutics, New York, New York, USA; b Pulmonary Biology, University of Cincinnati College of Medicine, Cincinnati, Ohio, USA; c Human Microbiology Institute, New York, New York, USA

**Keywords:** *Candida glabrata*, *Pneumocystis*, antifungal resistant

## Abstract

An urgent need exists for new antifungal compounds to treat fungal infections in immunocompromised patients. The aim of the current study was to investigate the potency of a novel antifungal compound, MYC-053, against the emerging yeast and yeast-like pathogens Candida glabrata, Candida auris, Cryptococcus neoformans, and Pneumocystis species. MYC-053 was equally effective against the susceptible control strains, clinical isolates, and resistant strains, with MICs of 0.125 to 4.0 μg/ml. Notably, unlike other antifungals such as azoles, polyenes, and echinocandins, MYC-053 was effective against Pneumocystis isolates, therefore being the only synthetic antifungal that may potentially be used against Pneumocystis spp., Candida spp., and Cryptococcus spp. MYC-053 was highly effective against preformed 48-h-old C. glabrata and C. neoformans biofilms, with minimal biofilm eradication concentrations equal to 1 to 4 times the MIC. Together, these data indicated that MYC-053 may be developed into a promising antifungal agent for the treatment and prevention of invasive fungal infections caused by yeasts and yeast-like fungi.

## Abstract (precis)

An urgent need exists for new antifungal compounds to treat fungal infections in immunocompromised patients. The aim of the current study was to investigate the potency of a novel antifungal compound, MYC-053, against the emerging yeast and yeast-like pathogens Candida glabrata, Candida auris, Cryptococcus neoformans, and Pneumocystis species.

## INTRODUCTION

In the last decade, invasive fungal infections caused by non-albicans Candida species and other less-common emerging yeasts, such as Cryptococcus spp., have become the leading cause of mortality in immunocompromised individuals (1–4).

Thus, Candida glabrata has emerged as the most common non-albicans Candida causative agent of invasive fungal infection, including the cases of hospital-acquired bloodstream infections in the United States in patients with an aberrant immune response (5–9).

Antifungal resistance among fungi causing invasive fungal infections represents a clinical challenge due to the limited classes of antimycotics available (polyenes, azoles, and echinocandins) (10). The spread of multidrug-resistant strains of C. glabrata in the United States, i.e., those displaying resistance to at least two classes of antifungal drugs, is consistently associated with increased mortality, as described in recent studies (11–13). Notably, resistance to echinocandins is also increasing among C. glabrata isolates, with reported resistance rates of 3% to 12% in different countries (14, 15).

Another global health care concern is the emerging multidrug-resistant pathogenic species Candida auris (16, 17). Unlike most other Candida spp., this fungus is commonly transmitted within health care facilities (18–20). Moreover, the drug resistance rate of C. auris exceeds that of C. glabrata, with over 41% of isolates reportedly resistant to at least two antifungal classes (18).

Cryptococcus neoformans is another opportunistic pathogen and an etiologic agent of cryptococcosis, a life-threatening infection in immunocompromised hosts (4). Although the rates of cryptococcosis have dropped substantially since the development of highly active antiretroviral therapy, the mortality of HIV patients associated with cryptococcal meningitis remains high. One of the causes of treatment failure is the emergence of azole-resistant and -heteroresistant mutants (21–23).

Pneumocystis species also affect immunocompromised hosts causing pneumocystis pneumonia (PcP) that, according to the Centers for Disease Control and Prevention, affects over 9% of hospitalized HIV patients in the United States (24) (http://www.cdc.gov/fungal/diseases/pneumocystis-pneumonia/statistics.html). Pneumocystis spp., originally classified as protozoa, are now classified as fungi but are not susceptible to antifungal drugs, being treated with sulfamethoxazole and pentamidine with a high mortality rate, from 5% to 40% (25, 26).

**FIG 1:**
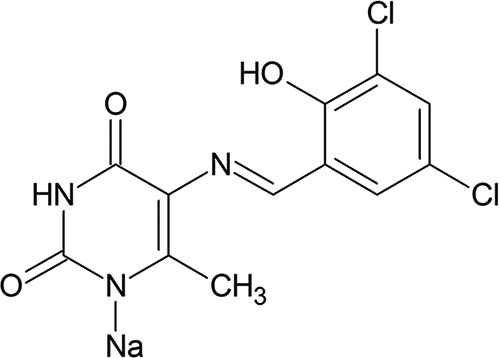
Chemical structure of MYC-053.

In this paper, we describe the fungicidal activity of a novel antifungal compound, MYC-053 {sodium 5-[1-(3,5-dichloro-2-hydroxyphenyl)methylideneamino]-6-methyl-1,2,3,4-tetrahydro-2,4-pyrimidinedionate} (Fig. 1), which is not related to any existing classes of antifungal agents and was investigated against planktonic and biofilm-forming Candida spp., Cryptococcus spp., and Pneumocystis spp. (27–31).

## RESULTS

### *In vitro* antifungal activity of MYC-053 against C. glabrata, C. auris, and C. neoformans.

The efficacy of MYC-053 against a panel of 20 C. glabrata strains (including seven fluconazole [FLC]-resistant and four caspofungin [CAS]-resistant strains), five C. auris strains, and 18 C. neoformans strains (including 12 FLC-resistant strains) was determined by the broth microdilution method (Table 1). MIC values of MYC-053 for C. glabrata strains varied from 1 to 4 μg/ml, while if applying a less restrictive endpoint criterion of 50% inhibitory concentration (IC_50_), values were in the low μg/ml range (0.125 to 0.5 μg/ml). C. auris and C. neoformans strains were also sensitive to this compound, with MICs of 1 to 4 μg/ml. Notably, the antifungal activity of MYC-053 against the susceptible strains, including the control C. glabrata ATCC 90030 and C. neoformans ATCC 90112 strains, was lower than that of FLC but higher than that of CAS. In contrast, while certain resistant clinical isolates exhibited reduced susceptibility to FLC and CAS, they were highly sensitive to the same concentrations of MYC-053 as the control strains (Table 1).

**TABLE 1:** Susceptibility and MIC and IC_50_ values of MYC-053 and other antifungal agents against *C. glabrata* and *C. auris*

Fungal species	Isolate no.	Susceptibility to^*a*^:		IC_50_ and MIC data by drug (μg/ml)^*b*^					
				MYC-053		FLC		CAS	
		FLC	CAS	IC_50_	MIC	IC_50_	MIC	IC_50_	MIC
*C. glabrata*	ATCC 90030	S	S	0.5	4	1	4	0.25	1
	CG1	I	NA	0.5	4	64	–	–	–
	CG2	S	NA	0.5	4	0.5	–	–	–
	CG3	I	NA	0.25	4	64	–	–	–
	CG4	S	NA	0.5	2	4	–	–	–
	CG5	S	NA	0.5	2	2	–	–	–
	CG6	I	NA	0.5	2	32	–	–	–
	CG7	I	NA	0.125	2	64	–	–	–
	CG8	I	NA	0.5	4	32	–	–	–
	CG9	I	NA	0.5	2	64	–	–	–
	CG10	R	NA	0.5	4	>64	–	–	–
	MR-V32	R	S	0.25	2	>64	>64	0.25	0.5
	MR-V35	R	R	0.5	4	>64	>64	4	4
	MR-V51	R	I	0.5	2	>64	>64	0.5	2
	MR-V16	R	R	0.125	1	>64	>64	4	8
	MR-V18	R	I	0.5	2	>64	>64	0.5	2
	MR-V19	R	R	0.5	4	>64	>64	2	2
	SS-V120	I	I	0.5	2	8	32	0.5	1
	SS-V114	S	S	0.25	2	2	8	0.125	0.25
	SS-V10	S	R	0.25	2	2	4	2	2
*C. auris*	CAU1	S	NA	1	4	2	–	–	–
	CAU2	S	NA	4	4	0.5	–	–	–
	CAU3	R	NA	4	4	>64	–	–	–
	V-2016-1	R	R	2	4	>64	>64	2	2
	V-2016-2	R	I	1	4	>64	>64	0.5	2
*C. neoformans*	ATCC 90030	S	NA	1	2	1	4	–	–
	CN1	R	NA	1	2	8	–	–	–
	CN2	S	NA	1	2	1	–	–	–
	CN3	R	NA	1	1	4	–	–	–
	CN4	R	NA	1	2	64	–	–	–
	CN5	R	NA	2	4	4	–	–	–
	CN6	R	NA	2	2	64	–	–	–
	CN7	R	NA	2	4	4	–	–	–
	CN8	S	NA	2	4	2	–	–	–
	CN9	S	NA	2	2	2	–	–	–
	CN10	S	NA	1	2	1	–	–	–
	RR-94	R	NA	0.5	2	64	>64	–	–
	RR-112	R	NA	2	2	8	>64	–	–
	RR-1025	R	NA	0.5	1	64	>64	–	–
	HR-30	R	NA	2	2	32	>64	–	–
	HR-02	R	NA	1	1	2	8	–	–
	SS-18	R	NA	2	4	2	>64	–	–
	SS-10	S	NA	1	1	1	8	–	–

a *Candida* spp. were considered susceptible (S) to FLC at an MIC of ≤8 μg/ml, intermediate (I) at an MIC of 8 to 64 μg/ml, and resistant (R) at an MIC of ≥64 μg/ml (50–52). *Candida* spp. were considered susceptible to CAS at an MIC of ≤0.25 μg/ml, intermediate at an MIC of 0.5 μg/ml, and resistant at an MIC of ≥1 μg/ml (35). Only potential breakpoints for FLC against *C. neoformans* were used, as follows: susceptible, ≤2 mg/liter; resistant, >2 mg/liter. NA, not available.

b –, not tested.

### *In vitro* antifungal activity of MYC-053 against Pneumocystis carinii and Pneumocystis murina.

The responses of Pneumocystis carinii and Pneumocystis murina to MYC-053 were evaluated by a cytotoxicity assay based on ATP-driven bioluminescence (32). The results, expressed as the IC_50_ after 24, 48, and 72 h of exposure to the drug, were assigned activity ranks based on the degree of reduction of ATP compared to the untreated controls (33, 34) (Table 2). The exposure of P. carinii to 1 μg/ml MYC-053 for 72 h resulted in a level of ATP reduction that was slightly lower than that of pentamidine and can be considered moderate activity. However, the increase in MYC-053 concentration to 10 μg/ml resulted in smaller amounts of ATP pools than with 1 μg/ml pentamidine. The inhibitory effect of MYC-053 against P. murina was higher than that against P. carinii. In this assay, MYC-053 demonstrated activity against P. murina comparable to that with pentamidine, with over 94.4% reduction of the ATP pool following 72 h of exposure, which is considered to indicate marked activity on the efficacy scale (34). Overall, MYC-053 effectively reduced the ATP content of both Pneumocystis species at microgram levels. The following IC_50_ values were calculated over 3 days of P. carinii exposure to MYC-053: 3.90 μg/ml at 24 h, 2.56 μg/ml at 48 h, and 1.61 μg/ml at 72 h. Against P. murina, the IC_50_ values were 3.30 μg/ml at 24 h, 1.50 μg/ml at 48 h, and 0.165 μg/ml at 72 h.

**TABLE 2:** IC_50_ values for MYC-053 for *P. carinii* and *P. murina* following different exposure times in the ATP assay

Drug, concn (μg/ml)	% reduction in ATP/media control^*a*^		
	24	48	72
*P. carinii*			
Ampicillin, 10	7.84	1.51	0
Pentamidine, 1	81.14	86.58	86,57
MYC-053, 50	96.81	97.61	99.21
MYC-053, 10	68.26	90.29	95.58
MYC-053, 1	14.95	11.08	26.77
MYC-053, 0.1	0	3.20	13.42
IC_50_	3.90 ± 2.0 μg/ml	2.56 ± 0.57 μg/ml	1.61 ± 1.72 μg/ml
P. murina			
Ampicillin, 10	2.86	0.26	0
Pentamidine, 1	92.07	97.70	98.12
MYC-053, 50	97.84	98.89	98.77
MYC-053, 10	76.56	98.51	97.92
MYC-053, 1	1.082	42.11	94.42
MYC-053, 0.1	0	0	27.82
IC_50_	3.30 ± 0.19 μg/ml	1.50 ± 0.13 μg/ml	0.165 ± 0.06 μg/ml

a Results represent the means from the 3 experiments which each contained three technical replicates.

### Activity of MYC-053 against C. glabrata and C. neoformans biofilms.

**FIG 2:**
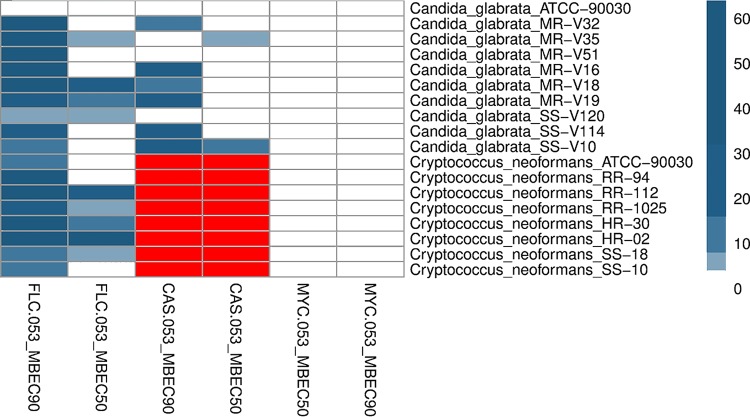
Heatmap and cluster analysis of MYC-053, FLC, and CAS against 48-h-old *C. glabrata* and *C. neoformans* biofilms expressed as MBEC_50_ or MBEC_90_, in multiples of MIC. The MIC values were ordered by hierarchical clustering using the Euclidean distance method and are represented by a heatmap, with the intensity indicated by a color code (dark blue, MIC; light blue, 8× the MIC; white, ≥64 times the MIC). *C. glabrata* strains that were not tested in this assay and *C. neoformans* strains that were not tested against CAS are highlighted in red.

The antibiofilm effects of MYC-053, FLC, and CAS on preformed 48-h-old C. glabrata biofilms and the effects of MYC-053 and FLC on cryptococcal biofilms were evaluated (Table 3). Preformed biofilms were exposed to drugs provided at concentrations equal to 1 to 64 times their MICs. MYC-053 significantly reduced the CFU of preformed biofilms of both C. glabrata and C. neoformans after 24 h of incubation, starting at a concentration of 1× the MIC. MYC-053 at a concentration of 1× the MIC decreased the number of viable fungi in all strains by more than 50%; this value was recorded as the 50% minimum biofilm eradication concentration (MBEC_50_). Moreover, MYC-053 was the only drug that showed MBEC_90_ values equal to 1 to 4 times its MIC. In the assay, higher relative concentrations of FLC and CAS were required to kill yeasts in preformed biofilms than concentrations of MYC-053. The MBEC_50_ and MBEC_90_ values of FLC and CAS against the tested preformed C. glabrata biofilms were equal to 4 to 64 times and 1 to 32 times their MICs, respectively. Similar data with high relative MBEC_50_ and MBEC_90_ of FLC required were obtained against C. neoformans biofilms (Fig. 2). CAS efficacy was not tested against C. neoformans biofilms in this assay, as this microorganism is known to be resistant both *in vitro* and *in vivo* to echinocandins.

**TABLE 3:** Susceptibility of 48-h-old *C. glabrata* biofilms to MYC-053, FLC, and CAS, expressed as multiples of MIC values

Fungal species	Isolate no.	MBEC data by drug (μg/ml)^*a*^					
		MYC-053		FLC		CAS^*b*^	
		MBEC_50_	MBEC_90_	MBEC_50_	MBEC_90_	MBEC_50_	MBEC_90_
*C. glabrata*	ATCC 90030	1	2	4	4	1	4
	MR-V32	1	2	4	>64	4	16
	MR-V35	1	1	8	>64	8	4
	MR-V51	1	2	4	>64	2	2
	MR-V16	1	2	4	>64	4	32
	MR-V18	1	4	32	>64	2	16
	MR-V19	1	2	16	32	4	32
	SS-V120	1	1	8	8	2	4
	SS-V114	1	4	4	32	4	32
	SS-V10	1	1	4	16	16	32
*C. neoformans*	ATCC 90030	1	2	2	16	–	–
	RR-94	1	4	4	>64	–	–
	RR-112	1	4	32	>64	–	–
	RR-1025	1	1	8	32	–	–
	HR-30	1	1	16	64	–	–
	HR-02	1	2	64	>64	–	–
	SS-18	1	2	8	16	–	–
	SS-10	1	1	4	16	–	–

a Results represent the means from 3 experiments, which each contained three technical replicates.

b –, not tested.

## DISCUSSION

In the current study, we described a novel antifungal drug candidate, MYC-053, which exhibited a high level of antimicrobial activity against C. glabrata, C. auris, C. neoformans, and Pneumocystis spp. *in vitro* that are well-known causes of morbidity in immunocompromised patients, being characterized by growing antibiotic resistance (35–42).

Importantly, the MIC experiment revealed that MYC-053 exerted a pronounced cidal effect against resistant fungal isolates at concentrations identical to the ones killing susceptible control fungal strains. These data correspond well with the notion that MYC-053 is a representative of a novel chemical class of antifungal agents; it is not relevant to the existing antifungal agents whose use is frequently characterized by cross-resistance (43). Notably, MYC-053 was effective against C. auris, which is often multidrug resistant (44). Although we have only tested the activity of MYC-053 against five C. auris strains, low standard error of the mean (SEM) values in the assay suggested high precision of the measurements, allowing us to determine the mean IC_50_ as 1 μg/ml and MIC as 4 μg/ml. Despite the fact that the MIC values of MYC-053 against C. auris were higher than those against C. glabrata, these values were nonetheless promising given the low susceptibility of certain tested strains to FLC and CAS, with MIC values over 64 μg/ml for these antifungals.

MYC-053 was also effective against C. neoformans, with MIC values starting at 1.0 μg/ml. These concentrations were dramatically different from the FLC MIC values. Although we did not test the sensitivity of C. neoformans strains against other azoles, it is known that this fungus is commonly cross-resistant to other antifungal agents of this class, including voriconazole (45, 46). Therefore, we propose that MYC-053 might be effective against other non-azole-resistant strains of C. neoformans.

This investigation also revealed that MYC-053 was effective against Pneumocystis spp., other yeast-like pathogens that are challenging to treat in immunocompromised patients. The anti-Pneumocystis activity of MYC-053 was promising since, despite being originally classed as protozoa, Pneumocystis spp. are now classified as fungi and continue to be generally treated with antibacterial and antiprotozoan medications (32, 47). The determination of ATP levels for the assessment of MYC-053 activity against Pneumocystis spp. constitutes a highly sensitive assay enabling a reduction in the number of tested organisms (33). The activity of MYC-053 was considered marked and was comparable to the activity of pentamidine against P. murina at the 72-h time point. To the best of our knowledge, MYC-053 is the first new synthetic compound that can be potentially used against Pneumocystis spp., Candida spp., and Cryptococcus spp.

We also revealed that MYC-053 was highly effective against 48-h-old preformed fungal biofilms. At a concentration equal to the MIC, MYC-053 caused a 50% reduction in the viable cell counts in all studied fungal biofilms. The MBEC_90_ values of MYC-053 were equal to 1 to 4 times the MIC values. Notably, the MIC/MBEC_50/90_ ratios of MYC-053 were significantly lower than those of the control antifungals FLC and CAS. In summary, MYC-053 was equally effective against sessile and planktonic nonresistant organisms and multiresistant clinical isolates.

Taken together, the results of the current study on the efficacy of MYC-053 against certain yeasts and yeast-like pathogens, including ones in a biofilm state, indicate the possibility of developing MYC-053 further into an antifungal drug candidate; however, it requires more *in vivo* research.

## MATERIALS AND METHODS

### Test substance and antimicrobials.

MYC-053 was synthesized by TGV-Therapeutics, Inc. (Wilmington, DE); FLC, CAS, and pentamidine were purchased from Sigma-Aldrich (St. Louis, MO) (Fig. 1).

### Fungal strains.

Forty-four fungal species were used in this study. C. glabrata CG1, CG2, CG3, CG4, CG5, CG6, CG7, CG8, CG9, and CG10, C. auris CAU1, CAU2, and CAU3, and C. neoformans CN1, CN2, CN3, CN4, CN5, CN6, CN7, CN8, CN9, and CN10 were obtained from the Fungus Testing Laboratory at the University of Texas Health Science Center (San Antonio, TX). C. glabrata MR-V32, MR-V35, MR-V51, MR-V16, MR-V18, MR-V19 SS-V120, SS-V114, and SS-V10, C. auris V-2016-1 and V-2016-2, and C. neoformans RR-94, RR-112, RR-1025, HR-30, HR-02, SS-18, and SS-10 were provided by V. Tetz (Human Microbiology Institute) from a private collection. P. carinii and P. murina were obtained from Melanie Cushion’s laboratory at the University of Cincinnati (Cincinnati, OH). The control strains were C. glabrata ATCC 90030 and C. neoformans ATCC 90112 (ATCC, Rockville, MD, USA). C. glabrata and C. auris isolates were subcultured on Sabouraud dextrose agar before testing (Oxoid Ltd., Basingstoke, UK).

### *In vitro* antifungal susceptibility testing.

Microdilution broth susceptibility testing was performed in duplicate according to the CLSI M27-A3 method in RPMI 1640 growth medium (Sigma-Aldrich) to determine the MIC values (48). Standard inoculum for yeast testing was 2.5 × 10^3^ CFU/ml. FLC and CAS were dissolved in dimethyl sulfoxide (DMSO; Sigma-Aldrich), whereas MYC-053 was dissolved in sterile water. The IC_50_ was defined as the lowest concentration of a drug that at which 50% growth inhibition was observed compared to the growth control. MIC was defined as the lowest concentration of the drug that resulted in no visual growth after 24 h of incubation at 35°C. Fungal isolates were categorized as susceptible, intermediate, or resistant according to the susceptibility breakpoints for antifungals based on CLSI criteria (49–52). The MIC experiments were performed in triplicate.

### *In vitro* P. carinii and P. murina ATP assays.

MYC-053 was diluted directly in the culture medium (0.1, 1, 10, and 50 μg/ml). The culture medium was RPMI 1640 containing 20% horse serum, 1% minimum essential medium (MEM)-vitamin solution, 1% MEM-nonessential amino acids (NEAA), 200 U/ml penicillin, and 0.2 mg/ml streptomycin (Sigma-Aldrich). The medium alone and medium containing 10 μg/ml ampicillin (Sigma-Aldrich) were the negative controls. Medium supplemented with 1 μg/ml pentamidine isethionate was the positive control. Cryopreserved and characterized P. carinii strains isolated from rat lung tissue and P. murina strains isolated from mouse lung tissue were distributed into triplicate wells of 48-well plates (final volume, 500 μl; final concentrations, 5 × 10^7^ nuclei/ml for P. carinii and 5 × 10^6^ nuclei/ml for P. murina). The controls and diluted compounds were added to the cultures and incubated at 35°C under 5% CO_2_. After 24, 48, and 72 h, 10% of the well volume was removed, and ATP content was determined using the ATP-Lite luciferin-luciferase assay (PerkinElmer, Waltham, MA). The ATP-associated luminescence was determined using a spectrophotometer (POLARstar Optima; BMG-Labtech, Germany). Each sample was examined microscopically on the final day of the assay to rule out the presence of bacteria. A quench control assay to determine compound interference in the luciferin/luciferase reaction was negative at all tested concentrations. Background luminescence was subtracted, and triplicate well readings were averaged. For each time point, the percent reduction in ATP content in all groups was calculated as follows: [ATP medium control − (ATP experimental/ATP medium control)] × 100. The IC_50_ was calculated using the INSTAT linear regression program (GraphPad Software, Inc., San Diego, CA). Each test was performed in triplicate.

### Effect of MYC-053 on preformed fungal biofilms.

A standardized C. glabrata or C. neoformans culture inoculum (200 μl; 5 × 10^5^ CFU/ml) in RPMI 1640 was added to each well of a 96-well round-bottom polystyrene tissue culture microtiter plate (Sarstedt, Nümbrecht, Germany) (48, 53). Following 48 h of incubation at 35°C, biofilm samples were washed twice with phosphate-buffered saline to remove nonadherent cells and then exposed for 24 h to 200 μl of RPMI 1640 containing MYC-053, FLC, or CAS at concentrations equal to 1, 2, 4, 8, 16, 32, and 64 times their MICs. Untreated biofilms were used as negative controls. The number of viable fungi in the biofilm was determined by estimating the CFU number. Briefly, to estimate the CFU number, following exposure, well contents were aspirated to prevent antimicrobial carryover, and each well was washed three times with sterile deionized water. Biofilms were scraped thoroughly, with a particular attention to well edges (27). The well contents were aspirated and placed in 2 ml of isotonic phosphate buffer (0.15 M; pH 7.2), the total fungal CFU number was determined by serial dilution and plating on Sabouraud dextrose agar (SDA), and the culture was incubated for 24 h at 35°C. Data were log_10_ transformed and were compared with the data for untreated biofilms. The MBEC values of drugs were defined as the concentrations of drug that killed 50% (MBEC_50_) or 90% (MBEC_90_) of yeasts in preformed 48-h-old biofilms. All assays included three replicates and were repeated in three independent experiments.

### Statistical analysis.

The Mann-Whitney *U* test was used to evaluate the differences between antifungal-treated and control samples. Differences at a *P* value of <0.05 were considered significant. The nonparametric paired Wilcoxon signed-rank test was employed to analyze the pre- and postchallenge differences, and a *P* value of <0.05 was considered significant.

## References

[1] Kullberg BJ, Vasquez J, Mootsikapun P, Nucci M, Paiva J-A, Garbino J, Yan JL, Aram J, Capparella MR, Conte U, Schlamm H, Swanson R, Herbrecht R 2017 Efficacy of anidulafungin in 539 patients with invasive candidiasis: a patient-level pooled analysis of six clinical trials. J Antimicrob Chemother 72:2368–2377. doi:10.1093/jac/dkx116. 28459966 PMC5890675

[2] Sobel J 2006 The emergence of non-albicans Candida species as causes of invasive candidiasis and candidemia. Curr Infect Dis Rep 8:427–433. doi:10.1007/s11908-006-0016-6. 17064635

[3] Taur Y, Cohen N, Dubnow S, Paskovaty A, Seo S 2010 Effect of antifungal therapy timing on mortality in cancer patients with candidemia. Antimicrob Agents Chemother 54:184–190. doi:10.1128/AAC.00945-09. 19884371 PMC2798557

[4] Rodríguez-Cerdeira C, Arenas R, Moreno-Coutiño G, Vásquez E, Fernández R, Chang P 2014 Systemic fungal infections in patients with human immunodeficiency virus. Actas Dermosifiliogr 105:5–17. doi:10.1016/j.adengl.2012.06.032. 23107866

[5] Pfaller M, Diekema D 2007 Epidemiology of invasive candidiasis: a persistent public health problem. Clin Microbiol Rev 20:133–163. doi:10.1128/CMR.00029-06. 17223626 PMC1797637

[6] Colombo AL, de Almeida Júnior JN, Slavin MA, Chen SC, Sorrell TC 2017 Candida and invasive mould diseases in non-neutropenic critically ill patients and patients with haematological cancer. Lancet Infect Dis 17:e344–e356. doi:10.1016/S1473-3099(17)30304-3. 28774702

[7] Hachem R, Hanna H, Kontoyiannis D, Jiang Y, Raad I 2008 The changing epidemiology of invasive candidiasis. Cancer 112:2493–2499. doi:10.1002/cncr.23466. 18412153

[8] Lockhart S, Wagner D, Iqbal N, Pappas P, Andes D, Kauffman C, Brumble L, Hadley S, Walker R, Ito J, Baddley J, Chiller T, Park BJ 2011 Comparison of in vitro susceptibility characteristics of Candida Species from cases of invasive candidiasis in solid organ and stem cell transplant recipients: Transplant-Associated Infections Surveillance Network (TRANSNET), 2001 to 2006. J Clin Microbiol 49:2404–2410. doi:10.1128/JCM.02474-10. 21562099 PMC3147873

[9] Pfaller M, Neofytos D, Diekema D, Azie N, Meier-Kriesche H, Quan S, Horn D 2012 Epidemiology and outcomes of candidemia in 3648 patients: data from the Prospective Antifungal Therapy (PATH Alliance) registry, 2004–2008. Diagn Microbiol Infect Dis 74:323–331. doi:10.1016/j.diagmicrobio.2012.10.003. 23102556

[10] Reboli A, Shorr A, Rotstein C, Pappas P, Kett D, Schlamm H, Reisman A, Biswas P, Walsh T 2011 Anidulafungin compared with fluconazole for treatment of candidemia and other forms of invasive candidiasis caused by Candida albicans: a multivariate analysis of factors associated with improved outcome. BMC Infect Dis 11:261. doi:10.1186/1471-2334-11-261. 21961941 PMC3203347

[11] Alexander B, Johnson M, Pfeiffer C, Jiménez-Ortigosa C, Catania J, Booker R, Castanheira M, Messer S, Perlin D, Pfaller M 2013 Increasing echinocandin resistance in Candida glabrata: clinical failure correlates with presence of FKS mutations and elevated minimum inhibitory concentrations. Clin Infect Dis 56:1724–1732. doi:10.1093/cid/cit136. 23487382 PMC3658363

[12] Pfaller MA, Castanheira M, Lockhart SR, Ahlquist AM, Messer SA, Jones RN 2012 Frequency of decreased susceptibility and resistance to echinocandins among fluconazole-resistant bloodstream isolates of Candida glabrata. J Clin Microbiol 50:1199–1203. doi:10.1128/JCM.06112-11. 22278842 PMC3318516

[13] Farmakiotis D, Tarrand JJ, Kontoyiannis DP 2014 Drug-resistant Candida glabrata infection in cancer patients. Emerg Infect Dis 20:1833. doi:10.3201/eid2011.140685. 25340258 PMC4214312

[14] Healey K, Jimenez Ortigosa C, Shor E, Perlin D 2016 Genetic drivers of multidrug resistance in Candida glabrata. Front Microbiol 7:1995. 28018323 10.3389/fmicb.2016.01995 PMC5156712

[15] Thompson G III, Wiederhold N, Vallor A, Villareal N, Lewis J II, Patterson T 2008 Development of caspofungin resistance following prolonged therapy for invasive candidiasis secondary to Candida glabrata infection. Antimicrob Agents Chemother 52:3783–3785. doi:10.1128/AAC.00473-08. 18676885 PMC2565919

[16] Welsh RM, Bentz ML, Shams A, Houston H, Lyons A, Rose LJ, Litvintseva AP 2017 Survival, persistence, and isolation of the emerging multidrug-resistant pathogenic yeast Candida auris on a plastic health care surface. J Clin Microbiol 55:2996–3005. doi:10.1128/JCM.00921-17. 28747370 PMC5625385

[17] Tsay S, Kallen A, Jackson B, Chiller T, Vallabhaneni S 2017 Approach to the investigation and management of patients with Candida auris, an emerging multidrug-resistant yeast. Clin Infect Dis 66:306–311. 10.1093/cid/cix744 PMC5798232 29020224

[18] Lockhart S, Etienne K, Vallabhaneni S, Farooqi J, Chowdhary A, Govender NP, Colombo A, Calvo B, Cuomo CA, Desjardins CA, Berkow EL, Castanheira M, Magobo RE, Jabeen K, Asghar RJ, Meis JF, Jackson B, Chiller T, Litvintseva AP 2017 Simultaneous emergence of multidrug-resistant Candida auris on 3 continents confirmed by whole-genome sequencing and epidemiological analyses. Clin Infect Dis 64:134–140. doi:10.1093/cid/ciw691. 27988485 PMC5215215

[19] Piedrahita C, Cadnum J, Jencson A, Shaikh A, Ghannoum M, Donskey C 2017 Environmental surfaces in healthcare facilities are a potential source for transmission of Candida auris and other Candida species. Infect Control Hosp Epidemiol 38:1107–1109. doi:10.1017/ice.2017.127. 28693657

[20] Vallabhaneni S, Kallen A, Tsay S, Chow N, Welsh R, Kerins J, Kemble S, Pacilli M, Black S, Landon E, Ridgway J, Palmore T, Zelzany A, Adams E, Quinn M, Chaturvedi S, Greenko J, Fernandez R, Southwick K, Furuya E, Calfee D, Hamula C, Patel G, Barrett P, Lafaro P, Berkow E, Moulton-Meissner H, Noble-Wang J, Fagan R, Jackson B, Lockhart S, Litvintseva A, Chiller T 2016 Investigation of the first seven reported cases of Candida auris, a globally emerging invasive, multidrug-resistant fungus — United States, May 2013–August 2016. MMWR Morb Mortal Wkly Rep 65:1234–1237. doi:10.15585/mmwr.mm6544e1. 27832049

[21] Sionov E, Chang Y, Kwon-Chung K 2013 Azole heteroresistance in Cryptococcus neoformans: emergence of resistant clones with chromosomal disomy in the mouse brain during fluconazole treatment. Antimicrob Agents Chemother 57:5127–5130. doi:10.1128/AAC.00694-13. 23836187 PMC3811407

[22] Maligie M, Selitrennikoff C 2005 Cryptococcus neoformans resistance to echinocandins: (1,3)-glucan synthase activity is sensitive to echinocandins. Antimicrob Agents Chemother 49:2851–2856. doi:10.1128/AAC.49.7.2851-2856.2005. 15980360 PMC1168702

[23] Hanson K, Catania J, Alexander B, Perfect J 2017 Drug resistance in cryptococcosis. Antimicrob Drug Resist 2:1119–1140.

[24] Cushion MT 2011 Pneumocystis, p 1822–1835. *In* Versalovic J, Carroll KC, Funke G, Jorgensen JH, Landry ML, Warnock DW (ed), Manual of clinical microbiology, 10th ed American Society of Microbiology, Washington, DC.

[25] Clark A, Hemmelgarn T, Danziger-Isakov L, Teusink A 2015 Intravenous pentamidine for *Pneumocystis carinii/jiroveci* pneumonia prophylaxis in pediatric transplant patients. Pediatr Transplant 19:326–331. doi:10.1111/petr.12441. 25712369

[26] Lei G, Zhang C, Zimmerman M, Lee C 2015 Vitamin D as supplemental therapy for Pneumocystis pneumonia. Antimicrob Agents Chemother 60:1289–1297. doi:10.1128/AAC.02607-15. 26666941 PMC4775931

[27] Tetz G, Artemenko N, Tetz V 2009 Effect of DNase and antibiotics on biofilm characteristics. Antimicrob Agents Chemother 53:1204–1209. doi:10.1128/AAC.00471-08. 19064900 PMC2650517

[28] Tetz V, Tetz G 2010 Effect of extracellular DNA destruction by DNase I on characteristics of forming biofilms. DNA Cell Biol 29:399–405. doi:10.1089/dna.2009.1011. 20491577

[29] Sherry L, Rajendran R, Lappin D, Borghi E, Perdoni F, Falleni M, Tosi D, Smith K, Williams C, Jones B, Nile C, Ramage G 2014 Biofilms formed by Candida albicans bloodstream isolates display phenotypic and transcriptional heterogeneity that are associated with resistance and pathogenicity. BMC Microbiol 14:182. doi:10.1186/1471-2180-14-182. 24996549 PMC4105547

[30] Mitchell K, Taff H, Cuevas M, Reinicke E, Sanchez H, Andes D 2013 Role of matrix β-1,3 glucan in antifungal resistance of non-albicans Candida biofilms. Antimicrob Agents Chemother 57:1918–1920. doi:10.1128/AAC.02378-12. 23318790 PMC3623361

[31] Oh B, Shin J, Kim M, Sung H, Lee K, Joo M, Shin M, Suh S, Ryang D 2011 Biofilm formation and genotyping of Candida haemulonii, Candida pseudohaemulonii, and a proposed new species (Candida auris) isolates from Korea. Med Mycol 49:98–102. doi:10.3109/13693786.2010.493563. 20560864

[32] Pearson RD, Hewlett EL 1985 Pentamidine for the treatment of Pneumocystis carinii pneumonia and other protozoal diseases. Ann Intern Med 103:782. doi:10.7326/0003-4819-103-5-782. 3901852

[33] Cushion MT, Chen F, Kloepfer N 1997 A cytotoxicity assay for evaluation of candidate anti-Pneumocystis carinii agents. Antimicrob Agents Chemother 41:379–384. doi:10.1128/AAC.41.2.379. 9021195 PMC163717

[34] Cushion M, Collins M, Hazra B, Kaneshiro E 2000 Effects of atovaquone and diospyrin-based drugs on the cellular ATP of Pneumocystis carinii f. sp. carinii. Antimicrob Agents Chemother 44:713–719. doi:10.1128/AAC.44.3.713-719.2000. 10681344 PMC89752

[35] Pfaller M, Messer S, Moet G, Jones R, Castanheira M 2011 Candida bloodstream infections: comparison of species distribution and resistance to echinocandin and azole antifungal agents in intensive care unit (ICU) and non-ICU settings in the SENTRY Antimicrobial Surveillance Program (2008–2009). Int J Antimicrob Agents 38:65–69. doi:10.1016/j.ijantimicag.2011.02.016. 21514797

[36] Kumamoto C 2002 Candida biofilms. Curr Opin Microbiol 5:608–611. doi:10.1016/S1369-5274(02)00371-5. 12457706

[37] Krcmery V, Barnes A 2002 Non-albicans Candida spp. causing fungaemia: pathogenicity and antifungal resistance. J Hosp Infect 50:243–260. doi:10.1053/jhin.2001.1151. 12014897

[38] Perlin D 2007 Resistance to echinocandin-class antifungal drugs. Drug Resist Updat 10:121–130. doi:10.1016/j.drup.2007.04.002. 17569573 PMC2696280

[39] Lee W, Shin J, Uh Y, Kang M, Kim S, Park K, Jang H 2011 First three reported cases of nosocomial fungemia caused by Candida auris. J Clin Microbiol 49:3139–3142. doi:10.1128/JCM.00319-11. 21715586 PMC3165631

[40] Navalkele B, Revankar S, Chandrasekar P 2017 Candida auris: a worrisome, globally emerging pathogen. Exp Rev Anti Infect Ther 15:819–827. doi:10.1080/14787210.2017.1364992. 28783385

[41] Kovacs JA, Masur H 2000 Prophylaxis against opportunistic infections in patients with human immunodeficiency virus infection. N Engl J Med 343:672–672. 10805828 10.1056/NEJM200005113421907

[42] Sepkowitz K 1998 Effect of HAART on natural history of AIDS-related opportunistic disorders. Lancet 351:228–230. doi:10.1016/S0140-6736(05)78279-9. 9457088

[43] Panackal A, Gribskov J, Staab J, Kirby K, Rinaldi M, Marr K 2006 Clinical significance of azole antifungal drug cross-resistance in Candida glabrata. J Clin Microbiol 44:1740–1743. doi:10.1128/JCM.44.5.1740-1743.2006. 16672401 PMC1479212

[44] Sarma S, Upadhyay S 2017 Current perspective on emergence, diagnosis and drug resistance in *Candida auris*. Infect Drug Resist 10:155–165. doi:10.2147/IDR.S116229. 28652784 PMC5476417

[45] Mondon P, Petter R, Amalfitano G, Luzzati R, Concia E, Polacheck I, Kwon-Chung KJ 1999 Heteroresistance to fluconazole and voriconazole in Cryptococcus neoformans. Antimicrob Agents Chemother 43:1856–1861. doi:10.1128/AAC.43.8.1856. 10428902 PMC89380

[46] Sionov E, Chang Y, Garraffo H, Kwon-Chung K 2009 Heteroresistance to fluconazole in Cryptococcus neoformans is intrinsic and associated with virulence. Antimicrob Agents Chemother 53:2804–2815. doi:10.1128/AAC.00295-09. 19414582 PMC2704677

[47] Centers for Disease Control. 1989 Guidelines for prophylaxis against Pneumocystis carinii pneumonia for persons infected with human immunodeficiency virus. MMWR Morb Mortal Wkly Rep 262:1–9. 2524643

[48] Joffe L, Schneider R, Lopes W, Azevedo R, Staats C, Kmetzsch L, Schrank A, Del Poeta M, Vainstein M, Rodrigues M 2017 The anti-helminthic compound mebendazole has multiple antifungal effects against Cryptococcus neoformans. Front Microbiol 8:535. doi:10.3389/fmicb.2017.00535. 28400768 PMC5368277

[49] Clinical and Laboratory Standards Institute. 2008 Reference method for broth dilution antifungal susceptibility testing of yeasts, 3rd ed CLSI document M27-A3. Clinical and Laboratory Standards Institute, Wayne, PA.

[50] Pfaller M, Diekema D, Sheehan D 2006 Interpretive breakpoints for fluconazole and Candida revisited: a blueprint for the future of antifungal susceptibility testing. Clin Microbiol Rev 19:435–447. doi:10.1128/CMR.19.2.435-447.2006. 16614256 PMC1471993

[51] Pfaller M, Diekema D, Andes D, Arendrup M, Brown S, Lockhart S, Motyl M, Perlin D, CLSI Subcommittee for Antifungal Testing. 2011 Clinical breakpoints for the echinocandins and Candida revisited: integration of molecular, clinical, and microbiological data to arrive at species-specific interpretive criteria. Drug Resist Updat 14:164–176. doi:10.1016/j.drup.2011.01.004. 21353623

[52] Sudan A, Livermore J, Howard S, Al-Nakeeb Z, Sharp A, Goodwin J, Gregson L, Warn P, Felton T, Perfect J, Harrison T, Hope W 2013 Pharmacokinetics and pharmacodynamics of fluconazole for cryptococcal meningoencephalitis: implications for antifungal therapy and in vitro susceptibility breakpoints. Antimicrob Agents Chemother 57:2793–2800. doi:10.1128/AAC.00216-13. 23571544 PMC3716186

[53] Pierce C, Uppuluri P, Tristan A, Wormley F, Mowat E, Ramage G, Lopez-Ribot J 2008 A simple and reproducible 96-well plate-based method for the formation of fungal biofilms and its application to antifungal susceptibility testing. Nat Protoc 3:1494–1500. doi:10.1038/nport.2008.141. 18772877 PMC2741160

